# Induction of Apoptosis by Ethanolic Extract of *Corchorus olitorius* Leaf in Human Hepatocellular Carcinoma (HepG2) Cells via a Mitochondria-Dependent Pathway

**DOI:** 10.3390/molecules17089348

**Published:** 2012-08-03

**Authors:** Chia-Jung Li, Shang-Yu Huang, Meng-Yu Wu, Yu-Ching Chen, Shih-Fang Tsang, Jong-Ho Chyuan, Hsue-Yin Hsu

**Affiliations:** 1 Institute of Medical Sciences, Tzu Chi University, No. 701, Sec. 3, Zhongyang Rd., Hualien City, 970, Taiwan; Email: 97751101@stmail.tcu.edu.tw; 2 Department of Life Sciences, Tzu Chi University, No. 701, Sec. 3, Zhongyang Rd., Hualien City, 970, Taiwan; 3 School of Medicine, Tzu Chi University, No. 701, Sec. 3, Zhongyang Rd., Hualien City, 970, Taiwan; 4 Department of Anatomy, Tzu Chi University, No. 701, Sec. 3, Zhongyang Rd., Hualien City, 970, Taiwan; 5 Crop Improvement Section, Hualien District Agricultural Research and Extension Station, Council of Agriculture, Executive Yuan, No. 150, Sec. 3, Gian Rd., Gian Village, Gian Township, Hualien County, 973, Taiwan

**Keywords:** *Corchorus olitorius* L., HepG2 cells, mitochondria, apoptosis

## Abstract

*Corchorus olitorius* L., is a culinary and medicinal herb, widely used as a vegetable in several countries in Asia. Many studies have shown that *C. olitorius* contains several antioxidants and exhibits anti-inflammatory and anti-proliferative activities in various *in vitro* and *in vivo* settings. Recently, *C. olitorius* has been approved for its antitumor activity; however, the underlying molecular mechanisms remain unclear. The goal of this study was to investigate the effects of ethanol extract of *C. olitorius* (ECO) on the growth of human hepatocellular carcinoma (HepG2) cells and gain some insights into the underlying mechanisms of its action. We found that HepG2 cells, treated with ECO for 24 h at a concentration higher than 12.5 μg/mL, displayed a strong reduction in cell viability, whereas normal FL83B hepatocytes were not affected. DNA fragmentation and nuclear condensation were evidenced by the increased subG1 population of ECO-treated HepG2 cells. ECO triggered the activation of procaspases-3 and -9 and caused the cleavage of downstream substrate, poly ADP-ribose polymerase (PARP), followed by down-regulation of the inhibitor of caspase-activated DNase (ICAD) signaling. Moreover, the increased release of cytochrome *c* from mitochondria with decreased membrane potential demonstrated the apoptosis induced through the caspases cascade. Our findings indicated that ECO might be effective against hepatocellular carcinoma through induction of apoptosis via mitochondria-dependent pathway.

## 1. Introduction

Food plants, including fruits, vegetables, and spices are the primary sources of naturally occurring nutrients essential for human health [1,2]. Due to their health benefits, vegetables and fruits have become popular among consumers and the number of medicinal plants being used in healthcare and as food has increased worldwide [3]. These emerging edible plants contain several classes of phytochemicals that have been extensively studied for their properties *in vitro* and *in vivo* [4]. Phytochemicals with antioxidative, anti-inflammatory, antibacterial, antimutagenic, and anticarcinogenic properties are extremely attractive potential agents for preventing or treating diseases in humans [5].

Cancer is a leading cause of death worldwide, accounting for millions of death each year. Previous studies have reported that the intake of antioxidant-rich foods has several health benefits, helping prevent cancer, cardiovascular diseases, diabetes, and other oxidative stress-related chronic diseases [6]. The current hypothesis is that the highly reactive and bioactive phytochemical antioxidants present in these foods are mediators of this protective effect. The phytochemicals found in plant-based foods also have other biological properties, which are neither correlated with their antioxidant property *in vivo* nor with their role as gene expression modulators [1,7]. Due to the lack of curative anticancer drugs, some patients use these plant-derived nutrients in alternative or complementary traditional chemotherapy and/or radiotherapy [8]. Therefore, the need for new therapeutic options has prompted many researchers to evaluate the efficacy of compounds found in fruits, vegetables, herbs, and spices as potential anticancer agents [9].

*Corchorus olitorius* L. (COL) is an edible plant used as a popular seasonal vegetable for soup in Taiwan. It has a rigid fiber used as the material for making gunny bags. The young leaves of COL are rich in calcium, potassium, phosphate, iron, ascorbic acid, carotene, and other nutrients, and contain a large amount of mucilageous polysaccharides [10,11]. Recent studies have shown that compounds such as carotenoids, flavonoids, and vitamin C isolated from the leaves of COL exhibit significant antioxidative activity [10,11]. In addition, the leaves are reported to have ethnomedicinal importance as a demulcent and febrifuge [12], and also possess anti-inflammatory, analgesic, antitumor, and antimicrobial activities [13,14].

In order to develop more effective approaches for the prevention and treatment of hepatocellular carcinoma (HCC), new therapeutic strategies with less toxicity are needed. In the present study, we analyzed the effects of ethanol extract of COL (ECO) on cell viability in human HCC (HepG2) cells and investigated the mechanisms underlying its anti-proliferative activity.

## 2. Results and Discussion

### 2.1. Cytotoxicity of ECO on HepG2 and FL83B Cells

**Figure 1:**
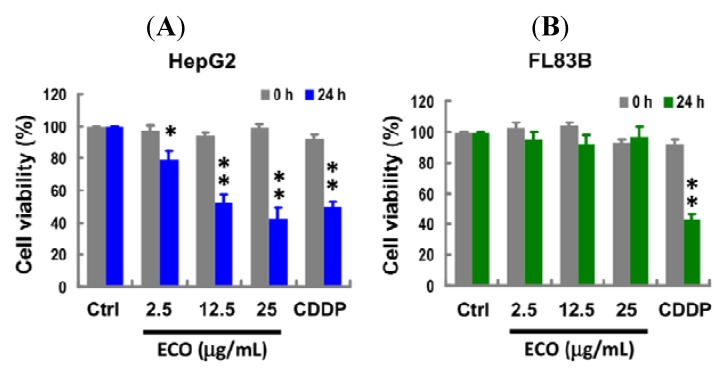
Effect of ECO on cell viability in HepG2 and FL83B cells. HepG2 cells (**A**) and FL83B cells (**B**) were treated with ECO at different concentrations for 24 h. Cell viability measured by the MTT assay was normalized to the control cells and data were represented as the mean ± SD. * *p* < 0.05 and ** *p* < 0.01 as compared to the control. Cells treated with CDDP at a concentration of 7.5 μg/mL were used as the positive control for the induction of cell death.

Both HepG2 and FL83B cells were used to examine the antiproliferative effects of ECO. Data shown in Figure 1 indicated that ECO treatment significantly inhibited the proliferation of HepG2 cells in a dose-dependent manner. Cell viability in ECO-treated HepG2 cells was observed at 24 h to be 52.2% ± 2.2% and 79.2% ± 3.2% for 12.5 and 2.5 μg/mL of ECO, respectively (Figure 1A). Hence, there was no significant cytotoxicity effect of ECO on FL83B cells at any treated concentration (Figure 1B). Cisplatin (CDDP) was used to compare with the antiproliferative effect of ECO in this study for its well known chemotherapeutic effect on the induction of cell death. The cytotoxic effect of CDDP (7.5 μg/mL) on normal hepatocytes was higher than that on HepG2 cells. The different cytotoxicity of ECO on HepG2 and FL83B cells indicated the specific antiproliferative effect of ECO on HepG2 cells. To further evaluate the cytotoxicity effect of ECO, the IC_50_ concentration of 12.5 μg/mL observed in this evaluation was further used for ECO treated on HepG2 cells at different time points.

### 2.2. ECO-Induced Apoptosis in HepG2 Cells

**Figure 2:**
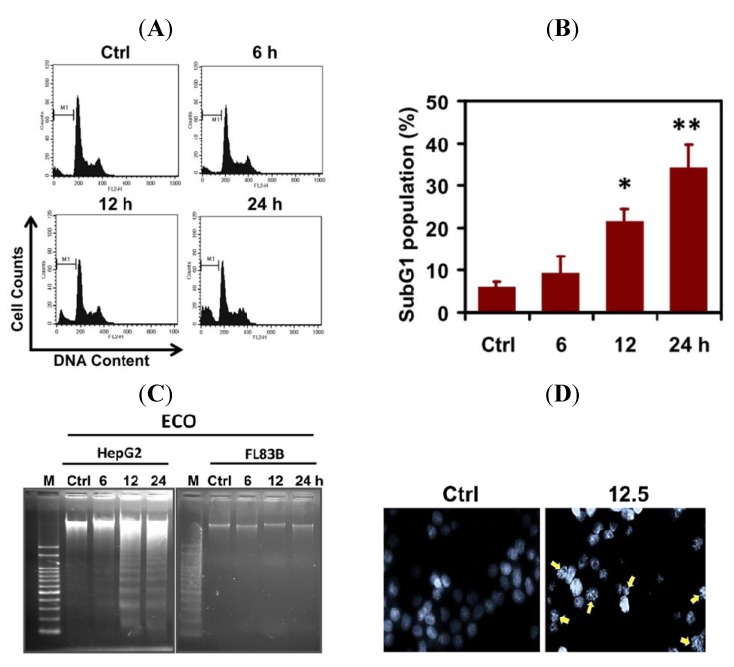
Apoptosisinduced byECO in HepG2 cells. (**A**) HepG2 cells were incubated with ECO (12.5 μg/mL) and the cell cycle was analyzed by PI staining and the representative cell cycle profiles were shownat 0, 6, 12, and 24 h. (**B**) Cells at subG1 phase were quantified after treatment with ECO. The histogram shows the mean ± SD of cells at subG1 phase for at least 3 independent experiments. * *p* < 0.05 and ** *p* < 0.01 as compared to untreated cells. (**C**) DNA fragmentation in HepG2 and FL83B cells treated with 12.5 μg/mL of ECO. (**D**) Nuclear condensation (shown by arrows), represented by DAPI staining, in HepG2 cells treated with ECO for 24 h.

To investigate how ECO induced the cytotoxicity of HepG2 cells, 12.5 μg/mL of ECO was used to treat cells and the effect on cell cycle progression was analyzed by FACS and PI staining (Figure 2A). We found that ECO induced cell death as shown by DNA fragmentation at subG1 phase in HepG2 cells (Figure 2A,B). Cells at subG1 phase were significantly increased by treatment of ECO for 12 and 24 h (* *p* < 0.05 and ** *p* < 0.01). Cell population at subG1 phase was 34.5 ± 5.2% at 24 h, as compared to 9.5 ± 3.8% and 21.5 ± 3.1% at 6 and 12 h, respectively. To confirm the cell death in ECO-treated HepG2 cells, we analyzed the DNA fragments appeared in cells according to the procedure for apoptotic DNA extraction as described previously [15]. As shown in Figure 2C, the ladder pattern of DNA fragments was observed in ECO-treated HepG2 cells, but not in FL83B cells. In addition, DNA condensation which is a hallmark of apoptosis was observed in HepG2 cells treated with ECO for 12 h by DAPI staining (Figure 2D). The increased subG1 population of ECO-treated HepG2 cells at 24 h coincided with that of the cell viability assay. Furthermore, DNA fragmentation found in HepG2 cells treated with ECO for 12 h were consistent with the significantly increased accumulation of subG1 cell population, indicating the time point for severe DNA damages induced by ECO, leading to apoptosis.

### 2.3. Caspases-Mediated Apoptosis in ECO-Treated HepG2 Cells

Pathways leading to apoptosis involve extrinsic and intrinsic processes which are initiated by the activation of caspases in a sequential cascade of cleaved caspases. Activation of caspase-3 is known to be activated by caspase-9 which is mediated by the loss of mitochondrial membrane potential and the consequent release of cytochrome *c*.

**Figure 3:**
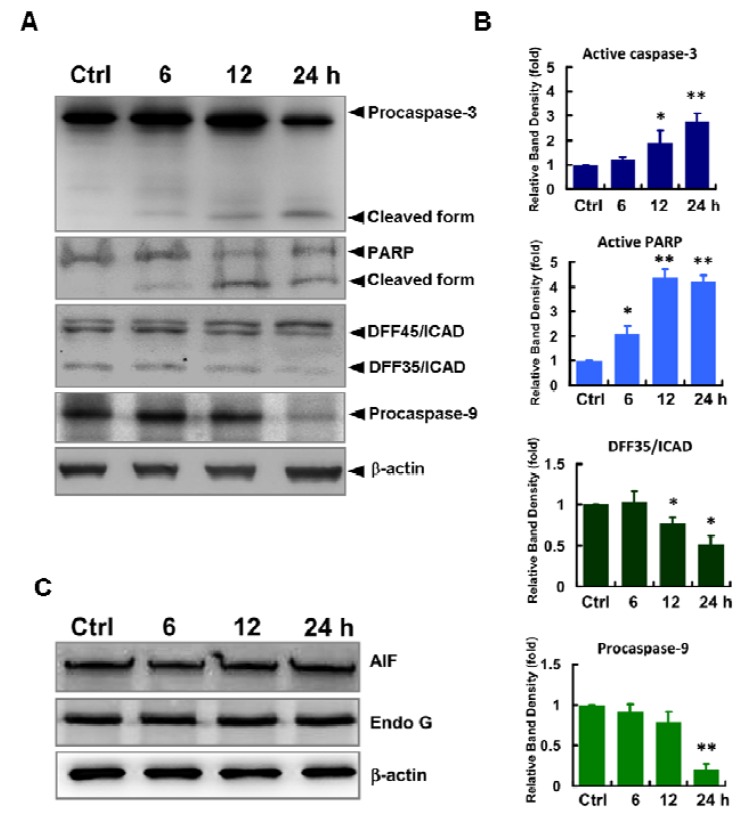
Expression of apoptotic proteins in ECO-treated cells. Proteins of HepG2 cells treated with 12.5 μg/mL of ECO were evaluated by western blot analysis. (**A**) Expression of caspases-9 and -3, PARP and DFF45/ICAD in ECO-treated HepG2 cells. (**B**) Quantified protein expressions of caspases-9 and -3, PARP and DFF45/ICAD in ECO-treated HepG2 cells. Activation of caspase-3 was quantified by the ratio of cleaved form/procaspase-3. The cleaved ICAD as shown by DFF35/ICAD from DFF45/ICAD in (**A**) was quantified by DFF35/ICAD. Protein levels were normalized respectively to β-actin and represented as folds of control. Data are represented by mean ± SD from three independent experiments. * *p* < 0.05 and ** *p* < 0.01 as compared to the vehicle controls. (**C**) Expression of AIF and EndoG in ECO-treated HepG2 cells.

To further elucidate the ECO-mediated cell death in HepG2 cells, we evaluated the expression of caspase-9, caspase-3 and the downstream substrates of caspase-3, poly(ADP-ribose) polymerases (PARP) and DNA fragmentation factor 45 (DFF45)/inhibitor of caspase-activated DNase (ICAD) (Figure 3A, B). In addition, apoptosis inducing factor (AIF) and endonuclease G (EndoG), proapoptogenic proteins released from mitochondria with increased membrane permeability were also evaluated in ECO-treated HepG2 cells (Figure 3C). ECO significantly decreased the expression of procaspase-9 and ICAD, whereas it increased the levels of cleaved caspase-3 and PARP in HepG2 cells after treating for 24 h. However, expression of AIF and endo G in ECO-treated HepG2 cells remained unchanged after treatment.

### 2.4. Mitochondria-Mediated Cell Death in ECO-Treated HepG2 Cells

**Figure 4:**
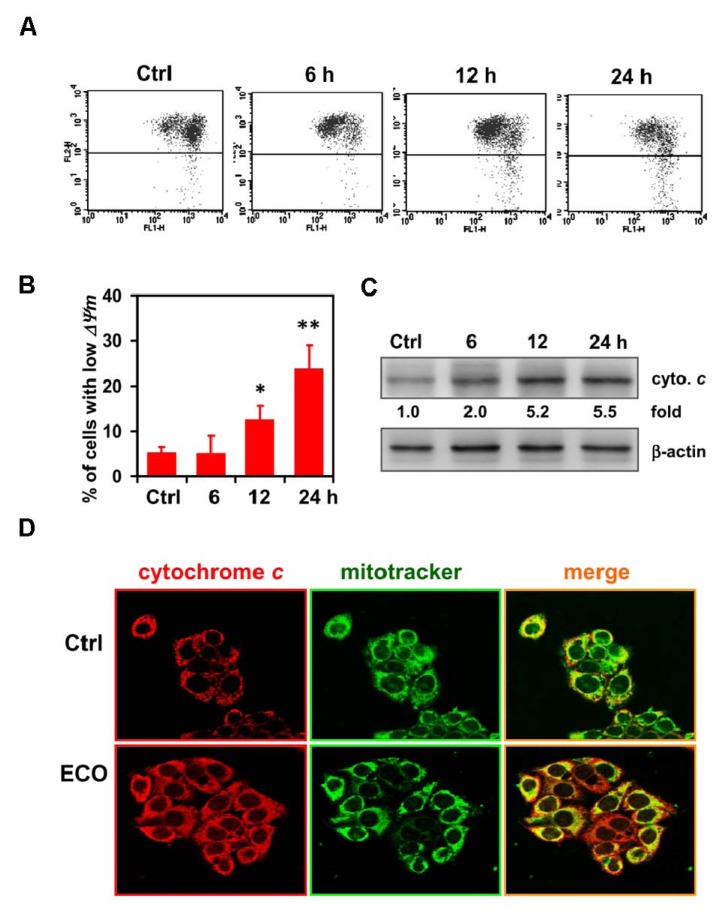
ECO-induced loss of mitochondrial membrane potential and the release of cytochrome *c*. (**A**) Mitochondrial membrane potential in ECO-treated HepG2 cells evaluated by JC-1 staining and flow cytometry analysis. JC-1 was used to trace the alteration of mitochondrial membrane potential. Representative FL1/FL2 profiles with green/red fluorescence are shown in ECO-treated HepG2 cells with concentration of 12.5 μg/mL. (**B**) Quantification of the JC-1 level with high green emission in the cells treated with ECO as shown in (**A**). Data were represented by mean ± SD. (**C**) Localization of cytochrome *c* in ECO-treated HepG2 cells. Cells were treated with ECO for 24 h, and the subcellular distribution of cytochrome *c* (stained by red fluorescence) was examined by immunostaining. MitoTracker (stained by green fluorescence) was used as a mitochondrial marker (original magnification, ×630).

Our results indicated that caspase-9, an initial caspase in the mitochondria-meidated apoptotic pathway, was shown to be activated in ECO-treated HepG2 cells. ECO-induced apoptosis in HepG2 cells was mediated through a pathway other than that of AIF and Endo G for their unchanged expression after treatments. Thus, we further investigated the effect of ECO on mitochondrial membrane potential (*ΔΨm*), which can be induced during the early stage of apoptosis, in HepG2 cells by using a fluorescence-based mitochondria-specific voltage-dependent dye, 5,5′,6,6′-tetrachloro-1,1′,3,3′-tetraethyl-benzimidazol-carbocyaniniodide (JC-1). JC-1 is a lipophilic cationic dye that enters the mitochondria in a concentration that is proportional to the membrane potential. It forms J-aggregates, which have a red fluorescent emission signal, when the membrane potential is high, whereas it is a monomer, with a green fluorescent signal, at low membrane potential. The percentage of cells in the high-green and low-red regions was 23.8% at 24 h of ECO treatment, as compared to 5.1% and 12.5% at 6 h and 12 h, respectively (Figure 4A,B ). It indicated that loss of *ΔΨm* in ECO-treated HepG2 cells was time-dependent. Additionally, we examined the subcellular localization of cytochrome *c* to evaluate its release from mitochondria after ECO treatment by immunostaining. As shown in Figure 4C, cytochrome *c* accumulated in the cytosol was observed in ECO-treated cells at 24 h. These data suggested that the cytotoxic effect of ECO on HepG2 cells was mediated via a mitochondria-dependent apoptotic pathway.

### 2.5. Discussion

Natural products, including food plants, provide rich sources of compounds that can be used for anticancer drug discovery [16]. COL has been widely used as an edible and medicinal plant in India and the Philippines. Traditionally, COL leaves have been used for their demulcent and diuretic properties, as a febrifuge and tonic, and in the treatment of chronic cystitis, gonorrhea, fever, and pain [17]. Primeval use of COL in deliberating diseases indicated that it exhibits several health benefit potentials for development as a health food or drug. In the present study, ECO exhibited anti-cancer effect on HepG2 cells at a concentration as low as non-toxic to the normal FL83B hepatocytes. We found that ECO induced cell death in HCC HepG2 cells was concentration-dependent. Cells at subG1 phase were increased according to the treated time, hence the significantly increased subG1 population at 12 h was consistent with that of DNA fragmentation in ECO-treated HepG2 cells. Nuclear condensation shown by shrinkage of nuclei and chromatin aggregation was found with the appearance of DNA fragmentation. 

Many factors mediating apoptosis converge to activate the critical effector, caspase-3, which is considered as one of the key proteases in the caspases cascade in mammalian cells [18,19]. Caspase-3 exists as a 32-kD inactive precursor in the cytoplasm and is cleaved and activated by the activation of its upstream caspase, caspase-9, during apoptosis. The activation of caspase-3 by ECO in HepG2 cells was significant, and increased in a time-dependent manner. Following caspase-3 activation, several proteins including PARP and ICAD are involved [20]. Activation of caspases and cleavage of PARP by caspases especially caspase-3 are the hallmarks of apoptosis [21]. In the present study, the activation of caspase-3 lead to increased cleavage of PARP and downregulation of ICAD, indicating the caspase-3-dependent pathway played a major role in ECO-induced apoptosis in HepG2 cells. Caspase-dependent apoptosis involves the mitochondrial pathway, death receptor pathway, and endoplasmic reticulum pathway [22]. To elucidate the regulatory mechanism of ECO-mediated apoptosis in HepG2 cells, we performed further experiments to explore that involved in the interaction of ECO with caspase-3.

Mitochondria are particularly affected in the early apoptotic process and are thought to act as central coordinators of cell death [18]. Mitochondrial dysfunction induces opening of the mitochondrial permeability transition pores, dissipation of *ΔΨm*, and release of apoptogenic proteins such as cytochrome *c*, AIF and EndoG [23]. AIF and EndoG are believed to play a key role in the regulation of caspase-independent cell death [24]. In this study, the activation of caspase-9, shown by the significant decrease of procaspase-9, demonstrated the role of mitochondrial pathway in ECO-mediated apoptosis in HepG2 cells. In addition to the loss of mitochondrial membrane potential, ECO increased the expression of cytochrome *c* in HepG2 cells, which was proposed to be induced by the formation of transport channels in the outer mitochondrial membrane [25]. Release of cytochrom *c* from mitochondria is an important trigger for activation of caspases. It showed that ECO gave significant disruption to mitochondrial membrane potential and only the increased level of cytochrome *c*, whereas non-significant alterations in the levels of AIF and EndoG, was observed. The identical expression of AIF and EndoG at different treated courses was yet enough to demonstrate their role in ECO-induced apoptosis in HepG2 cells.

**Figure 5:**
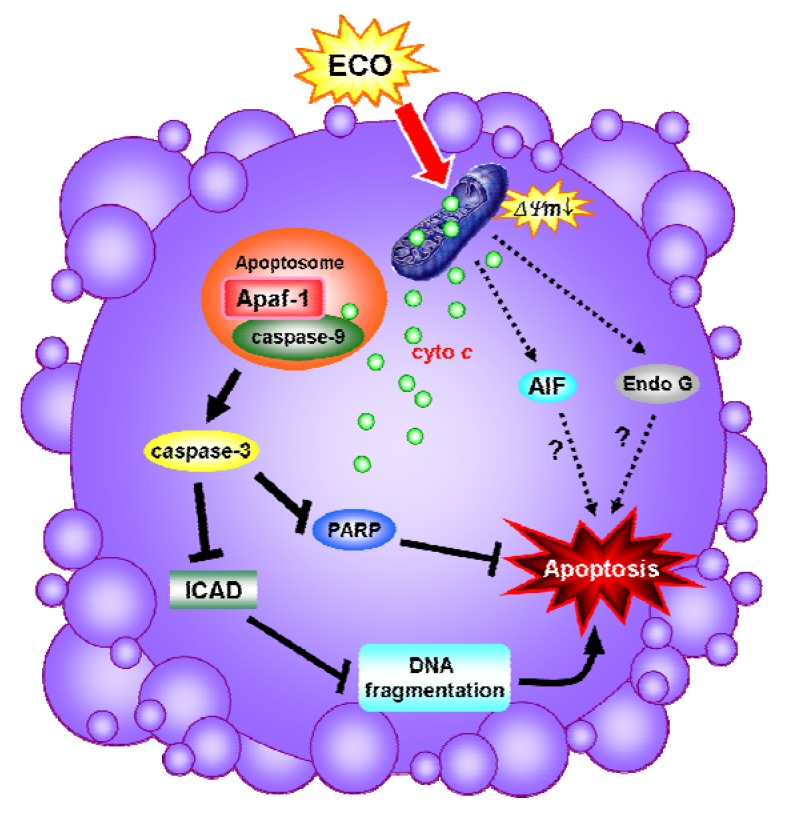
Diagram of ECO-induced apoptosis in HepG2 cells. ECO induced a loss of mitochondrial membrane potential which facilitated the exclusion of AIF, Endo G and cytochrome *c* from mitochondria. The formation of apoptosomes by cytochrome *c* activated caspase-9 which subsequently activated the caspase-3. PARP and ICAD, the downstream substrates of caspase-3, was cleaved or downregulated by the activated caspase-3, respectively, and thus induces DNA fragmentations, leading to apoptosis.

Phytol, a side chain of chlorophylls, and monogalactosyl-diacylglycerol which were reported to be antitumor promoters, had been identified in leaves of COL [26]. Previous studies suggested that the cytotoxicity of phytol on cancer cells was due to an induction of apoptosis [27]. Monogalactosyl-diacylglycerol was shown to inhibit the activities of mammalian DNA polymerases including repair-related DNA polymerase β and induce severe apoptosis in gastric cancer cells with IC_50_ values less than 50 μg/mL [28], a concentration higher than that used for ECO in this study. Moreover, the inhibitor role of monogalactosyl-diacylglycerol on replicative DNA polymerases was evidenced to selectively suppress the growth of several human cancer cell lines [29]. Thence, apoptosis in ECO-treated HepG2 cells through caspase-dependent mitochondrial pathway may be induced partially by phytol and monogalactosyl-diacylglycerol. Interestingly, damages to normal FL83B hepatocytes were not obvious under the effective concentrations of ECO used for HepG2 cells. In contrast to the activation of caspases, release of mitochondrial apoptogenic proteins, and DNA fragmentation followed by the cleavage of PARP and downregulated ICAD in ECO-treated HepG2 cells, absolution of ECO-induced apoptosis was explicit in FL83B cells (Figure 5). The differential cytotoxicity of ECO on HepG2 and FL83B cells was speculated to be due to the different regulatory mechanisms against ECO-induced stress in cells. However, it remains to be elucidated in the subsequent experiment. Due to its non-toxicity to normal hepatocytes at concentrations with anti-cancer effects on HCC HepG2 cells, the practicality of COL in development of health food or drug for complementary medicine will be greatly increased. 

## 3. Experimental

### 3.1. Plant Material

COL was cultivated and authenticated at the Hualien District Agricultural Research and Extension Station Council of Agriculture, Executive Yuan (HARES, Hualien, Taiwan). During June to September of the years 2008 and 2009, when the plants were about 1.5 m in height, young leaves branching from the stem were harvested in the morning. They were minced and subjected to a sequential extraction process with 95% ethanol for soaking after washing and air-drying at room temperature. Ethanol extracted supernatant of COL was filtered, centrifuged and evaporated to dryness using a rotatory evaporator under reduced pressure to obtain the crude mass. The crude extract (ECO) was then stored at −20 °C until required and dissolved in DMSO for treatments.

### 3.2. Cell Culture

The human HCC HepG2 cells and normal FL83B hepatocytes, purchased from Bioresource Collection and Research Center, Hsinchu, Taiwan, were grown in DMEM, commented with 10% heat-inactivated fetal bovine serum, 2 mM L-glutamine, 0.1 M sodium bicarbonate, penicillin (100 U/mL) and streptomycin (100 mg/mL). Cultured cells were maintained at 37 °C in a humidified atmosphere with 5% CO_2_.

### 3.3. Cell Viability Assay

ECO preserved at −20 °C was diluted with DMSO to 100 mg/mL before a series of dilutions with medium to prepare the tested concentrations. The cytotoxicity of ECO on cells was assessed using the 3-(4,5-dimethylthiazol-2yl)-2,5-biphenyltetrazolium bromide (MTT, Sigma Chemical Co., St. Louis, MO, USA) assay. HepG2 and FL83B cells were collected, suspended in medium, counted and plated in 96-well plates at a density of 6 × 10^3^ cells per well in 200 μL of culture medium. After incubation for 24 h with ECO, 100 μL of freshly prepared MTT reagent (0.5 mg/mL in culture medium) was added to each well and the plates were incubated for about 4 h at 37 °C at which point the formazan crystals were dissolved in 100 μL of DMSO and absorbance at 570 nm was measured with an ELISA reader (Bio-tek Instruments, Winooski, VT, USA). Each treatment was tested eight times in at least three independent experiments. 

### 3.4. Cell Cycle Analysis

Cells were seeded, treated with ECO for 24 to 48 h, and fixed overnight with 70% ethanol at −20 °C. For cell cycle analysis, cells were washed twice with PBS, and resuspended in 100 μL of propidium iodide (PI) solution (20 μg/mL PI, 0.1 mg/mL RNase A, and 0.1% Triton X-100 in PBS) for 30 min at room temperature in the dark. Distribution of cells with different DNA contents was analyzed on a FACS Calibur flow cytometer and CellQuest software (BD Biosciences, San Jose, CA , USA). A total of 1 × 10^4^ cells were analyzed per sample. Each experiment was performed in triplicate.

### 3.5. DNA Fragmentation Assay

The DNA fragmentation assay was performed as previously described [30].

### 3.6. Mitochondrial Membrane Potential

The measurement of mitochondrial transmembrane potential was performed using the JC-1-based assay (Invitrogen, Carlsbad, CA , USA). Cells were treated with ECO for 24 h, trypsinized, washed in PBS, resuspended in 0.5 mL culture medium containing 10 μg/mL JC-1 (Molecular Probes, Invitrogen, Carlsbad, CA, USA) staining dye, and incubated at 37 °C for 15 min. Subsequently, cells were washed twice with PBS, the cell pellet was resuspended, and analyzed by flow cytometry (FACS Calibur, BD Bioscience). A plot of red fluorescence (FL2) from living cells with intact mitochondrial membrane potential and green fluorescence (FL1) from cells with loss of mitochondrial membrane potential was recorded.

### 3.7. Western Blot

The Western blotting analysis was performed as described previously [30].

### 3.8. Immunofluorescence Labeling of Cytochrome *c*

HepG2 cells, treated with ECO for 24 h, were washed with PBS, and incubated with 200 nM MitoTracker Green (Molecular Probes, Invitrogen, Carlsbad, CA , USA) for 45 min at 37 °C. To determine the subcellular localization of cytochrome *c*, cells were washed twice and fixed with 4% paraformaldehyde in PBS, and subsequently permeabilized with 0.2% Triton X-100 on ice for 5 min. After washing with PBS twice, cells were incubated in blocking solution (PBS containing 20% goat serum) for 30 min at room temperature and then incubated overnight at 4°C with anti-cytochrome *c* antibody (ICON-GeneTex, Taipei, Taiwan). Cells were then washed with PBS and incubated with rhodamine-conjugated goat anti-rabbit secondary antibody at 1:200 dilution for 45 min. Cells were visualized and imaged with a confocal laser scanning microscope (Leica Inc., TCS-SP2, Mannheim, Germany). 

### 3.9. Statistical Analyses

Quantification of all experiments was conducted using a densitometer (Personal Densitometer SI, Molecular Dynamics, Sunnyvale, CA, USA) for statistical analyses. Data presented are the mean ± SD from at least 3 independent experiments. In all experiments, significance values (*p <* 0.05) were calculated with a Student’s t-test after one-way analysis of variance.

## 4. Conclusions

To our knowledge, this is the first study to demonstrate that ethanol extract of *C. olitorius* exhibits anti-proliferative effects on HepG2 cells by inducing apoptosis through mitochondria- and caspase-mediated pathways. Due to the distinct effect of ECO on HepG2 and normal FL83B hepatocytes, *C. olitorius* is suggested to be an edible plant with potential for the development of chemopreventive agents for human cancers.
